# Can non-tumorous liver tissue serve as a reliable reference measure for [^18^F]FDG-PET-CT after unilobar ^90^Y glass radioembolization in patients with colorectal liver metastases?

**DOI:** 10.1186/s13550-025-01320-9

**Published:** 2025-09-26

**Authors:** Li Shen Ho, Manon N. G. J. A. Braat, Guus Bol, Maarten. L. J. Smits, Marnix G. E. H. Lam, Arthur J. A. T. Braat

**Affiliations:** 1 Radiology and Nuclear Medicine, University Medical Center Utrecht, Heidelberglaan 100, Utrecht, The Netherlands; 2 Medical Oncology, University Medical Center Utrecht, Utrecht, The Netherlands; 3 Division of Hematology/Oncology, Department of Medicine, University of California, San Francisco, CA USA; 4 Nuclear Medicine, Netherlands Cancer Institute, Amsterdam, The Netherlands

**Keywords:** FDG-PET, Radioembolization, SIRT, PERCIST

## Abstract

**Background:**

Effect of radiation on [^18^F]FDG-uptake in non-tumorous liver tissue after radioembolization is sparsely investigated, despite the liver being the most common reference for assessment of metabolic response (e.g., PET Response Criteria In Solid Tumors). The study aims to evaluate changes in mean standardized uptake value corrected for lean body mass in treated and untreated non-tumorous liver after unilobar radioembolization with ^90^Y-glass microspheres in patients with colorectal liver metastases.

**Results:**

Eighteen patients were included. Median mean standardized uptake value corrected for lean body mass in the treated lobe increased from baseline 2.88 (range 2.24–3.81) to posttreatment 2.98 (range 2.53–3.53). In the untreated lobe, the median mean standardized uptake value corrected for lean body mass changed from 2.95 (range 2.23–4.02) to 2.88 (range 2.39–3.71), respectively. The median mean standardized uptake value corrected for lean body mass of the ratio treated/untreated liver changed from 0.96 (range .77–1.23) to 1.00 (range .88–1.26) after treatment (p = 0.147)). The correlation for the ratio (mean standardized uptake value corrected for lean body mass in treated liver divided by mean standardized uptake value corrected for lean body mass in untreated liver) before and after radioembolization was nonsignificant. No trend was seen between [^18^F]FDG-uptake and treated or untreated non-tumorous liver absorbed dose.

**Conclusions:**

This study shows no significant increase in [^18^F]FDG-uptake in both treated and untreated non-tumorous liver in patients with liver metastases of colorectal carcinoma, three months after lobar ^90^Y-glass microsphere radioembolization. This non-significant increase suggests that using background SUL in the non-tumorous liver (treated and untreated) is reliable and appropriate as a reference to assess metabolic response three months after unilobar radioembolization.

**Supplementary Information:**

The online version contains supplementary material available at 10.1186/s13550-025-01320-9.

## Introduction

Colorectal cancer is the third most common cancer type worldwide, with more than 1.9 million patients in 2022 [1] and about 25% of patients will develop distant metastases, most commonly to the liver [2], significantly impacting prognosis [3]. Standard treatment is curative surgical resection when feasible or palliative systemic treatment in inoperable cases [4]. However, non-surgical locoregional liver-directed treatments are becoming more prevalent with the availability of more advanced techniques [2, 5, 6]. Radioembolization successfully reduces tumor burden inpatients with liver metastases of colorectal carcinoma [7]. However, the effects and extent of radiation-induced changes in non-tumorous liver tissue remain unclear. This raises concerns whether the standardized uptake value (SUV) in non-tumorous liver tissue remains a reliable reference for [^18^F]FDG-PET-CT response assessment post-radiation treatment.

Current standard practice evaluates effectiveness primarily using CT according to the Response Evaluation Criteria In Solid Tumors version 1.1 [8, 9]. However, [^18^F]FDG-PET-CT is increasingly recognized as a valuable tool for assessing treatment response in colorectal liver metastases, as it may provide earlier indications for treatment efficacy, by revealing metabolic changes that precede anatomical response in liver metastases of colorectal carcinoma post-radioembolization [10]. The value of [^18^F]FDG-PET-CT during therapy effect assessment and prediction of prognosis was underlined by Bijlstra et al. [11]. The PET Response Criteria in Solid Tumors (PERCIST) [12] is specifically designed for metabolic tumor response assessment on [^18^F]FDG-PET-CT. It proposes the mean standardized uptake value (SUV_mean_) corrected for lean body mass (SUL_mean_) in the non-diseased liver parenchyma as quality control measure. Therefore, PERCIST ensures [^18^F]FDG-PET comparability across different timepoints and establishes a threshold to define target lesions.

The extent of metabolic changes throughout the liver after radioembolization remains inadequately defined. Specifically, it is unclear whether changes in SUL are confined to the treated area and might result from radiation-induced inflammation, or more diffuse throughout the whole liver, as a radiation hepatitis-like presentation. Recent findings indicate that [^18^F]FDG-uptake in the non-treated liver parenchyma increased significantly following radioembolization with ^90^Y-resin microspheres [13]. The explanation for these findings remains largely unknown. The authors suggest that sinusoidal obstruction syndrome may be a plausible explanation, as sinusoidal obstruction syndrome may lead to passive tracer stasis due to endothelial cell injury and peliotic changes. However, the authors were unable to differentiate between patients receiving (sequential) whole liver Or unilobar treatment, since 23 Of the 26 included patients underwent whole liver treatment.

This exploratory study aims to assess if SUL_mean_ in the liver can be used as a reference for therapy response assessment after lobar ^90^Y-glass microspheres radioembolization in patients with colorectal liver metastases.

## Methods

### Patient selection

This is a retrospective monocenter study. All patients with liver metastases of colorectal carcinoma treated with ^90^Y-glass microspheres (Therasphere; Boston Scientific, Marlborough, Massachusetts, USA) radioembolization between March 2015 and April 2024 were screened for eligibility. The following inclusion criteria were applied: (1) patients with liver metastases of colorectal carcinoma; (2) radioembolization with ^90^Y-glass microspheres and (3) segmental/unilobar treatment. Exclusion criteria were: (1) (sequential) whole liver treatment, (2) missing [^18^F]FDG-PET-CT prior and/or after treatment or post treatment ^90^Y-PET, (3) follow-up scan longer or shorter than three months (12 ± 2 weeks) posttreatment,(4) prior (extended) hemihepatectomy, and (5) previously treated with holmium microsphere radioembolization. The local institutional medical ethics committee waived the need for informed consent for this retrospective analysis.

### Data collection

Patient demographics (i.e., sex and mean age) were collected from the electronic case report forms. Clinical data including tumor side, oncologic surgery, onset-time of liver metastases, mutation status (i.e., BRAF, KRAS and MSS/MSI) and type Of previously administered systemic therapy were documented. Furthermore, pretreatment and 3-months (± 1 month) posttreatment biochemical data was collected including alkaline phosphatase (ALP), aspartate aminotransferase (AST),alanine aminotransferase (ALT) and bilirubin.

### [18F]FDG-PET-CT imaging protocols

[^18^F]FDG-PET-CT scans were deemed either assessable or suboptimal according to the PERCIST criteria, i.e.,injection-to-imaging time difference of ≤ 15 min between baseline and follow-up injection-to-acquisition time ≥ 50 or ≤ 70 min during either baseline or follow-up scan or; difference in SUL in the non-tumorous liver between baseline and follow-up scan ≤ 0.3 SUL units or ≤ 20% [14]. All scans were performed on either Biograph40 mCT, Biograph VISION 600 or Biograph128 V600 Edge (Siemens Healthcare, Erlangen, Germany). Patients received intravenous [^18^F]FDG activity Of 2.0 MBq/kg after 6-h fasting. Blood glucose level was measured before tracer injection, to ensure it was below 11.1 mmol/L. The imaging parameters for the most used scanner (Biograph mCT) included a 3-dimensional acquisition technique with a 216 mm field Of view, 3 min per bed position, and Ordered subset expectation maximization iterative reconstruction including a Gaussian filter, 4 iterations and 21 subsets. The measurements were performed on image reconstructions according to EARL-criteria [15].

### Image analysis

All [^18^F]FDG-PET-CT scans were analyzed using Syngo.via (Siemens Healthcare, Erlangen, Germany). The liver was divided into three regions: segment 1, segments 2–4 (left hemiliver) and segments 5–8 (right hemiliver). Spheric volume of interests (VOI)s Of 3 cm in diameter were placed in the left and right liver region prior and after radioembolization. No VOI was placed in segment 1, as vascularization is variable. The preferred VOI location of both sides was in the peripheral area of the non-diseased liver, to obtain consistent SUL_mean_ measurements. In case of too extensive tumor invasion in one of the regions to accommodate the VOI, the patient was excluded. Liver lobes were labelled as treated or untreated lobe. Four VOIs were placed per patient: VOI_treated, prior_, VOI_untreated, prior_, VOI_treated, post_ and VOI_untreated, post_ (Supplemental Fig. 1). A cylindrical VOI Of 2 cm diameter was (semi-)automatically placed in the descending aorta without including the aortic wall to avoid elevated [^18^F]FDG-uptake due to atherosclerosis. The SUL_mean_, in accordance with PERCIST 1.0 [12], was calculated for all VOIs.

**Fig. 1:**
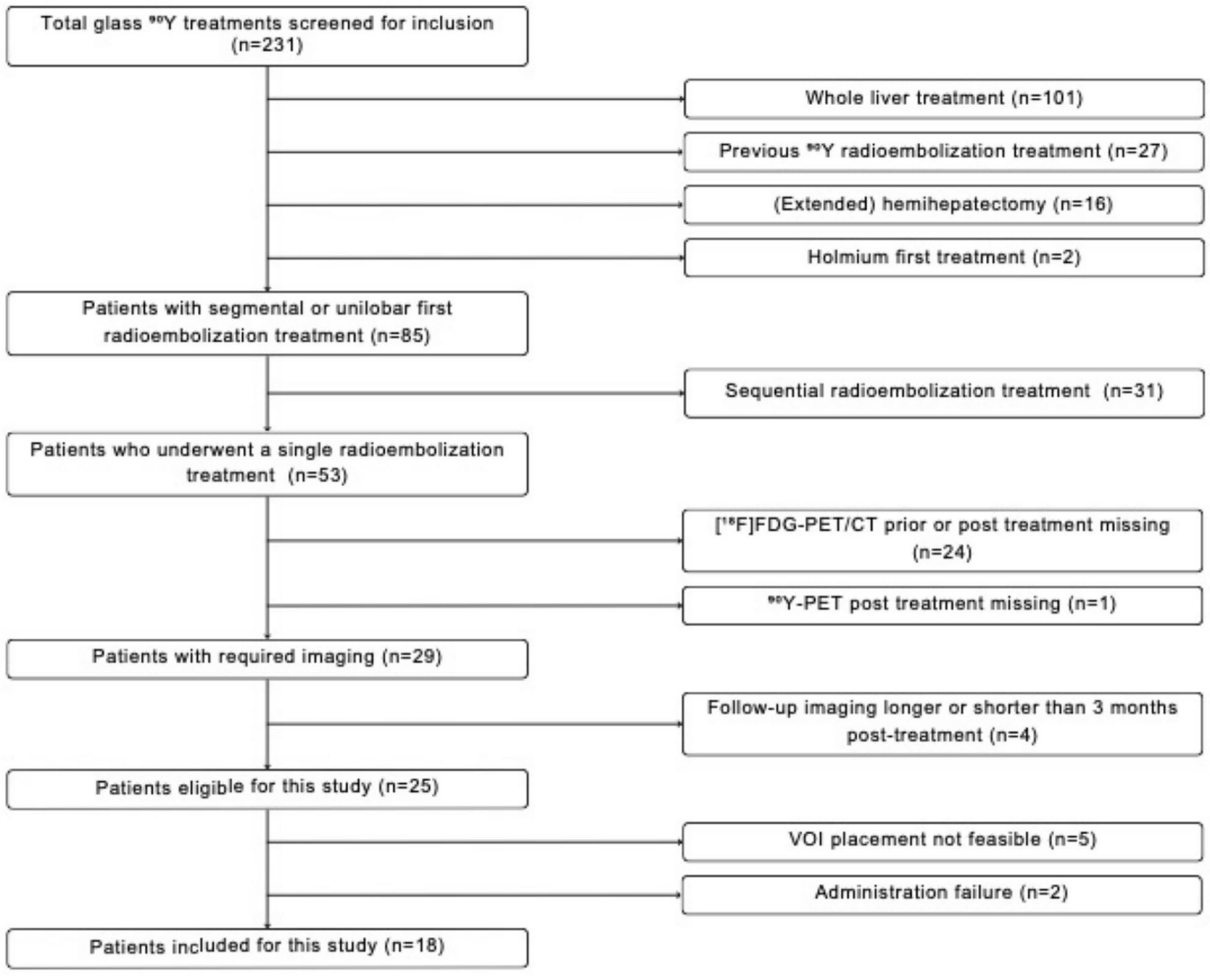
Flowchart of the included patients with liver metastases of colorectal carcinoma ^90^Y, Yttrium-90; VOI, volume of interest

### Dosimetry and volumetric assessment.

Dosimetric calculations were done using Simplicit90y (Mirada Medical, Denver, CO). Pretreatment portal-phase contrast-enhanced CT and posttreatment ^90^Y-PET-CT were semi-automated co-registered. In case of an absent portal-phase contrast-enhanced CT, the arterial phase contrast-enhanced CT was used, with knowledge of the disease on [^18^F]FDG-PET. VOIs of non-tumorous liver and tumor(s) on the contrast-enhanced CT were manually drawn. Non-tumorous liver was defined by subtracting tumor VOI from total liver VOI, and absorbed dose was calculated for the perfused non-tumorous liver and tumor VOIs. ^90^Y-PET-CT images were scaled to administered activity (corrected for measured residual activity).

### Statistical analyses

Descriptive statistics were used to explore baseline and treatment characteristics. To evaluate differences in SUL_mean_ prior and after radioembolization, a paired T-test was used. The paired t-test was also used to evaluate differences in blood glucose and reference blood pool on the scan day prior compared to after radioembolization. Additionally, boxplots were created to visualize the distribution of SUL_mean_ values prior and after treatment. Differences were considered statistically significant when p-value ≤ 0.05. The statistical analyses were performed using SPSS 25.0 (Statistical Package for Social Sciences, Chicago, IL, USA).

## Results

### Patient characteristics

A total Of 188 patients with 235 treatments from September 2009 until April 2024 were screened for eligibility, Of whom 18 patients (12 male; 6 female) met the inclusion criteria (Fig. 1). Clinical characteristics of the included patients are detailed in Table 1, highlighting that 11 patients did not have the primary tumor in situ at time Of radioembolization and 13 patients had synchronous liver metastases. Two patients did not receive systemic treatment before radioembolization. Most patients (82%) were KRAS/BRAF wildtype.

**Table 1:** Baseline characteristics of the included patients and, volumetric and dosimetric parameters of the included radioembolization treatments

	N = 18
**Sex** Female (%) Male (%)	6 (33%) 12 (67%)
**Mean age in years (range)**	73 (52–92)
**Primary tumor side** Left (%) Right (%) Rectal (%)	3 (17%) 9 (50%) 6 (33%)
**Primary in situ**	7 (39%)
**Liver metastases** Synchronous (%) Metachronous (%)	13 (72%) 5 (28%)
**Mutation status** BRAF^V600^ mutation, (unknown) KRAS mutation, (unknown) MSS, (unknown)	0 (2) 3 (1) 17 (1)
**Previous systemic therapy** Yes (%) No (%)	16 (89%) 2 (11%)
**Previous systemic therapies containing:** 5-FU (%) Oxaliplatin (%) Capecitabine (%) Irinotecan (%) Bevacizumab (%)	7 (39%) 13 (72%) 15 (83%) 6 (33%) 8 (44%)
**Median liver volume in cm**^**3**^** (range)**	1496 (911–2843)
**Median administered activity in GBq (range)**	2.8, (0.4–11.5)
**Median treated fraction in % (range)**	61 (9–84)
**Median perfused non-tumorous liver absorbed dose in Gy (range)**	107 (50–420)
**Median perfused tumor absorbed dose in Gy (range)**	241 (78–1098)

MSS = microsatellite stable; 5-FU = fluorouracil; GBq = gigabecquerel

### [^18^F]FDG-PET

Eight patients did not fully meet the PERCIST criteria and were therefore considered suboptimal (Table 2). For two patients, the timing of tracer administration was unknown, as the scans were performed at an external facility. Median time interval between pre- and post-treatment [^18^F]FDG-PET-CT was 124 days (range 98–138 days).

**Table 2:** PERCIST criteria fulfillment of the included patients

	Optimal	Suboptimal*
Difference in SUL in the non-tumorous liver between baseline and follow-up scan ≤ 20%	17	1
Difference in SUL in the non-tumorous liver between baseline and follow-up scan ≤ 0.3 SUL units	12	6
Injection-to-imaging time difference of ≤ 15 min between baseline and follow-up	17	1
Injection-to-acquisition time was ≥ 50 or ≤ 70 min during either baseline or follow-up scan	13	5
**Overall PERCIST criteria fulfillment**	**10**	**8**

^*^ Patients may be represented in more than one (sub)optimal category

Median SUL_mean_ in the treated lobe pretreatment (SUL_mean, prior_) was 2.88 (range 2.24–3.81) and 2.98 (range 2.53–3.53) posttreatment (SUL_mean, post_). For the untreated liver lobe these were 2.95 (range 2.23–4.02) pretreatment and 2.88 (range 2.39–3.71) posttreatment, respectively. Median ratio SUL_mean,treated_/SUL_mean,untreated_ pretreatment was 0.96 (range 0.77–1.23), and 1.00 (range 0.87–1.26) posttreatment. The mean differences between pretreatment and posttreatment in the treated and untreated lobe were nonsignificant, neither was the treated/untreated liver ratio (Table 3).

**Table 3:** Paired sample T-test of pretreatment and posttreatment SUL_mean_

	**Mean**	**SD**	**95% CI**	**Two-sided p-value**
${\mathbf{S}\mathbf{U}\mathbf{L}}_{\mathbf{m}\mathbf{e}\mathbf{a}\mathbf{n},\mathbf{t}\mathbf{r}\mathbf{e}\mathbf{a}\mathbf{t}\mathbf{e}\mathbf{d},\mathbf{p}\mathbf{r}\mathbf{i}\mathbf{o}\mathbf{r}}$**–**${\mathbf{S}\mathbf{U}\mathbf{L}}_{\mathbf{m}\mathbf{e}\mathbf{a}\mathbf{n},\mathbf{t}\mathbf{r}\mathbf{e}\mathbf{a}\mathbf{t}\mathbf{e}\mathbf{d},\mathbf{p}\mathbf{o}\mathbf{s}\mathbf{t}}$	-.08944	.34904	-.26302-.08413	.292
${\mathbf{S}\mathbf{U}\mathbf{L}}_{\mathbf{m}\mathbf{e}\mathbf{a}\mathbf{n},\mathbf{u}\mathbf{n}\mathbf{t}\mathbf{r}\mathbf{e}\mathbf{a}\mathbf{t}\mathbf{e}\mathbf{d},\mathbf{p}\mathbf{r}\mathbf{i}\mathbf{o}\mathbf{r}}$**–**${\mathbf{S}\mathbf{U}\mathbf{L}}_{\mathbf{m}\mathbf{e}\mathbf{a}\mathbf{n},\mathbf{u}\mathbf{n}\mathbf{t}\mathbf{r}\mathbf{e}\mathbf{a}\mathbf{t}\mathbf{e}\mathbf{d},\mathbf{p}\mathbf{o}\mathbf{s}\mathbf{t}}$	-.03167	.39393	-.16423-.22756	.737
$\frac{{\mathbf{S}\mathbf{U}\mathbf{L}}_{\mathbf{m}\mathbf{e}\mathbf{a}\mathbf{n},\mathbf{t}\mathbf{r}\mathbf{e}\mathbf{a}\mathbf{t}\mathbf{e}\mathbf{d},\mathbf{p}\mathbf{r}\mathbf{i}\mathbf{o}\mathbf{r}}}{{\mathbf{S}\mathbf{U}\mathbf{L}}_{\mathbf{m}\mathbf{e}\mathbf{a}\mathbf{n},\mathbf{u}\mathbf{n}\mathbf{t}\mathbf{r}\mathbf{e}\mathbf{a}\mathbf{t}\mathbf{e}\mathbf{d},\mathbf{p}\mathbf{r}\mathbf{i}\mathbf{o}\mathbf{r}}}-\frac{{\mathbf{S}\mathbf{U}\mathbf{L}}_{\mathbf{m}\mathbf{e}\mathbf{a}\mathbf{n},\mathbf{t}\mathbf{r}\mathbf{e}\mathbf{a}\mathbf{t}\mathbf{e}\mathbf{d},\mathbf{p}\mathbf{o}\mathbf{s}\mathbf{t}}}{{\mathbf{S}\mathbf{U}\mathbf{L}}_{\mathbf{m}\mathbf{e}\mathbf{a}\mathbf{n},\mathbf{u}\mathbf{n}\mathbf{t}\mathbf{r}\mathbf{e}\mathbf{a}\mathbf{t}\mathbf{e}\mathbf{d},\mathbf{p}\mathbf{o}\mathbf{s}\mathbf{t}}}$	-.04218	.11773	-.10072 -.01636	.147

SUL_mean_, mean standardized uptake value corrected for lean body mass; SD, standard deviation; CI, confidence interval

**Fig. 2:**
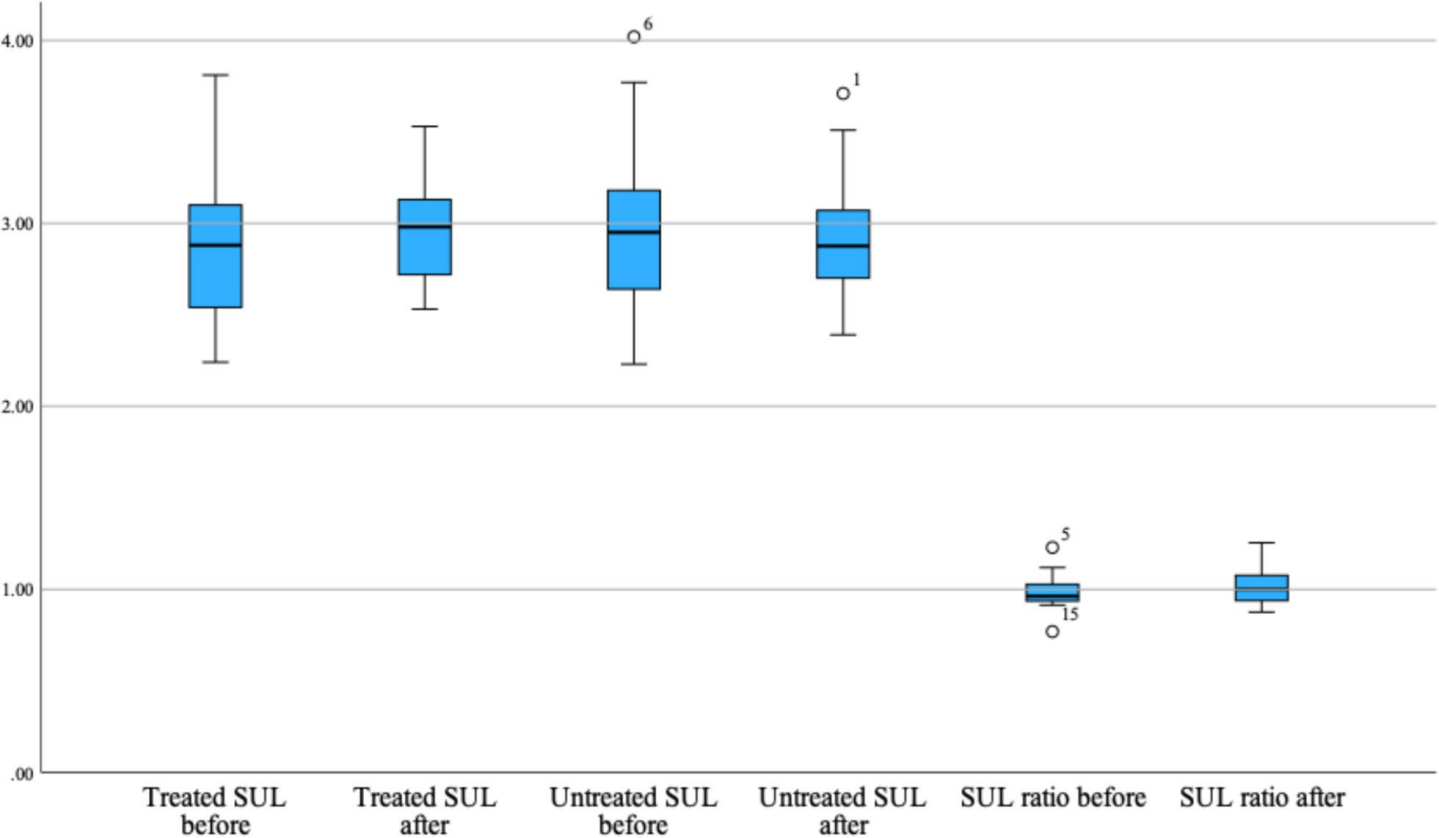
Boxplots of SUL_mean_ in the treated liver, untreated liver, and treated/untreated liver ratio before and after ^90^Y-glass radioembolization. Abbreviations SUL_mean_ = mean standardized uptake value corrected for lean body mass

In contrast, a positive correlation was observed between measured SUL_mean_ from pretreatment to posttreatment scans in both the treated (0.570, p = 0.013) and untreated lobe (0.563, p = 0.015). The correlation for the ratio of SUL_mean_ treated/untreated liver was not statistically significant (0.363, p = 0.139). Boxplots of the distribution of SUL_mean_ values before and after radioembolization treatment in the treated, untreated, or treated/untreated liver ratio remained similar across timepoints, with overlapping interquartile ranges (Fig. 2). Paired t-test showed no significant difference in blood glucose or reference blood pool SUL on the day of scanning between pretreatment and posttreatment measurements (p = 0.791 and p = 0.552, respectively).

### Volumetric and dosimetric parameters

The whole liver volume ranged from 911 to 2843 cm^3^, with a median Of 1496 cm^3^ (Table 1). Median Of 61% of the liver was treated (range 9–84%) with an administered activity ranging from 0.4 to 11.5 GBq. Dosimetric analysis revealed that the median perfused non-tumorous liver absorbed dose was 107 Gy (range 50–420 Gy), while the median perfused tumor absorbed dose was 241 Gy (range 78–1098 Gy). The analysis revealed no correlation between the absorbed dose in healthy liver tissue and changes in SUL_mean_ before and after ^90^Y-glass radioembolization (data not shown).

### Biochemical changes

Biochemical markers changed following treatment (Table 4). ALP increased, with a median change Of 30 U/L (range 93–656 U/L) and mean change Of 94.44 with a 95% confidence interval (CI) Of 0.01–1.88 (p = 0.048). Bilirubin increased with a median change Of 4 ηmol/L (range −5–15 ηmol/L) and mean change Of 3.76 with a 95% CI Of 0.77–6.75 (p = 0.017). Other parameters, including AST and ALT, were not statistically significant.

**Table 4:** Changes in mean biochemical markers (posttreatment minus pretreatment values) after radioembolization

	**Mean**	**SD**	**95% CI**	**Two-sided p-value**
Δ ALP	94.444	188.007	.95063–187.93826	.048 *
Δ AST	4.22222	35.91093	−13.63586 – 22.08031	.624
Δ ALT	8.00000	48.77560	−16.25553 – 32.25553	.496
Δ Bilirubin	3.76471	5.81517	.77482–6.75459	.017 *

SD, standard deviation; CI, confidence interval; Δ, difference; ALP, alkaline phosphatase; AST, aspartate transaminase; ALT, alanine transaminase.* Differences were considered statistically significant when p-value ≤ 0.05

## Discussion

No significant difference was found after lobar radioembolization compared to baseline in SUL_mean_ in both treated and untreated non-tumorous liver regions. The SUL_mean_ ratio between treated and untreated lobes did not differ posttreatment either. While dosimetry is critical for ensuring safety and effective radiation delivery, non-tumorous liver absorbed dose does not appear to evidently impact liver metabolism as reflected by SUL on [^18^F]FDG-PET-CT following ^90^Y-glass microsphere radioembolization.

The results of this study tend to support the retrospective study of Bienert et al. [16]. Their analysis included five patients and found no difference in [^18^F]FDG-uptake in the treated compared to the untreated non-tumorous liver tissue after ^90^Y-resin radioembolization. Although Nakahara et al. [17] described an increased [^18^F]FDG-uptake in their case report at 26 days after external beam radiation therapy, this finding normalized after 4 months. Therefore, it is possible that the [^18^F]FDG-uptake returned to its previous state by the time of the follow-up [^18^F]FDG-PET-CT scan in this analysis, three months after treatment. However, it should be noted that in contrast to external beam radiotherapy, which delivers a homogeneous radiation dose, radioembolization results in a heterogeneous dose distribution within the targeted liver. This variability may influence the timeline and pattern of metabolic changes observed on [^18^F]FDG-PET-CT. Furthermore, a mildly positive correlation was observed between the SUL_prior_ and SUL_post_ in both the treated and untreated liver parenchyma. This correlation suggests that in both lobes, there is a relationship between changes in liver metabolism and treatment. When correlating the ratio (SUL_mean_ in treated liver divided by SUL_mean_ in untreated liver) before and after radioembolization, the correlation was smaller and no longer statistically significant. This could indicate that while both lobes respond to unilobar radioembolization, the extent of metabolic changes in the treated/untreated liver varies among patients.

Comparison of [^18^F]FDG-uptake in the liver with previous reported data is challenging, because of different ways of quantification or correction of liver [^18^F]FDG-uptake. These variations include, e.g., use of different VOIs; use of SUV_max_, SUV_mean_ or TLG; or various correction methods, including body surface area (BSA), body weight or LBM (SUL). VOI size is of relevance as SUVs measured in a larger and fixed VOI are more reproducible and precise than using a smaller VOI [18]. Spheric VOIs with a diameter Of 3 cm are sufficient for measuring SUV_mean_ [19], similarly to the approach used in this study. SUL was chosen in this study, as this correction method is more consistent from patient to patient than body weight [20] or BSA [21]. In addition, this is not affected by clinical parameters, i.e., age, sex, blood glucose level or diabetes [22–24].

In a retrospective review by Viner et al. [20] including 116 patients, who had undergone an [^18^F]FDG-PET-CT for oncologic staging, the liver SUL_mean_ values had low interreader and intersite variability, regardless of VOI placement within the right liver lobe. The intraclass correlation coefficients (ICC) of SUL_mean_, adjusted for readers, were 0.986 (95%CI 0.98, 0.991; p = 0.0001) for reader 1 and 0.987 (95%CI 0.981, 0.991; p = 0.0001) for reader 2. When adjusted for location, the ICC for SUL_mean_ measurements by two readers were 0.986 (95%CI: 0.979–0.99) at the upper level and 0.98 (95%CI 0.971–0.986) at the lower level. This demonstrates that the method used in the current study, VOI placement in non-tumorous liver tissue, is a robust measurement with low interobserver variability. While automated VOI placement tools can enhance efficiency, their outputs should not be accepted without careful review. Reader experience by reporting physicians remains crucial when assessing liver uptake on [^18^F]FDG-PET-CT. Misplacement due to tumor involvement or artifacts may lead to inaccurate SUL measurements. In this study, all VOIs were manually reviewed and adjusted as needed. (Supplemental Fig. 1).

Prior received systemic therapy may introduce changes in SUV_mean_ measurements and therefore SUL, as was described in patients with lymphoma treated with chemotherapy, but that is also reported to resolve over time [25–27]. Taking the results Of this study and previous reports into consideration, the liver seems adequate as a reference at 3 months posttreatment for the assessment of metabolic response on [^18^F]FDG-PET-CT following radioembolization.

This study has several limitations. Firstly, although approximately 200 patients were screened for this study, only a limited number of patients met all study criteria, leading to a small sample size. This limitation does affect the statistical power and limits generalizability of the results. Secondly, the quantitative results of [^18^F]FDG-PET-CT depend On e.g., hardware, image acquisitions, reconstruction and analyses. As treatments and imaging data Over 15 years were included in the analyses, improvements in imaging techniques and PET scanners may introduce variability in SUL measurements [28]. However, in this monocenter study all imaging studies were performed with the same accredited image acquisition and reconstruction protocols (EARL). In addition, the [^18^F]FDG-PET-CT analyses were semi-automated and corrected by one researcher (LSH) under supervision of an experienced nuclear physician (AB, > 10 years experience). Therefore, we have limited the effect of technical differences. Thirdly, the sample size of this study was too small to correct for confounding factors, like previous systemic therapies and potential underlying liver disease (chemotherapy induced liver steatosis).

Future larger studies incorporating [^18^F]FDG-PET-CT before and after radioembolization should investigate metabolic changes Over time before the 3-month time-point and the influence of other factors, like systemic therapies. In addition, prospective studies should incorporate the use of [^18^F]FDG-PET-CT and PERCIST as response assessment after radioembolization, to further validate its use.

## Conclusion

This study shows no significant increase in [^18^F]FDG-uptake in both treated and untreated non-tumorous liver in patients with liver metastases of colorectal carcinoma, three months after lobar ^90^Y-glass microsphere radioembolization. This suggests that using non-tumorous liver as a reference seems reliable and appropriate for assessment of treatment response with [^18^F]FDG-PET-CT.

## Supplementary Information

Additional file 1.

## Data Availability

Not applicable.

## Abbreviations

[^18^F]FDG: Fluorine-18 fluorodeoxyglucose
^90^Y: Yttrium-90
ALP: Alkaline phosphatase
ALT: Alanine aminotransferase
AST: Aspartate aminotransferase
BRAF: B-Raf proto-oncogene
BSA: Body surface area
CI: Confidence interval
CT: Computed tomography
EANM: European association of nuclear medicine
EARL: EANM research ltd.
FDG: Fluorodeoxyglucose
FU: Fluorouracil
GBq: Gigabecquerel
Gy: Gray
ICC: Intraclass correlation coefficient
KRAS: Kirsten rat sarcoma viral oncogene homolog
LBM: Lean body mass
MBq: Megabecquerel
MSI: Microsatellite instability
MSS: Microsatellite stable
OSEM: Ordered subset expectation maximization
PERCIST: PET response criteria in solid tumors
PET: Positron emission tomography
RECIST: Response evaluation criteria in solid tumors
SD: Standard deviation
SUL: Standardized uptake value corrected for lean body mass
SULmean: Mean standardized uptake value corrected for lean body mass
SUV: Standardized uptake value
TLG: Total lesion glycolysis
VOI: Volume of interest
