# Supplementary figures and images for: Can non-tumorous liver tissue serve as a reliable reference measure for [18F]FDG-PET-CT after unilobar 90Y glass radioembolization in patients with colorectal liver metastases?
Source: EJNMMI Res. 2025 Sep 26;15:126. doi: 10.1186/s13550-025-01320-9 (PMC12474746; doi:10.1186/s13550-025-01320-9)

A

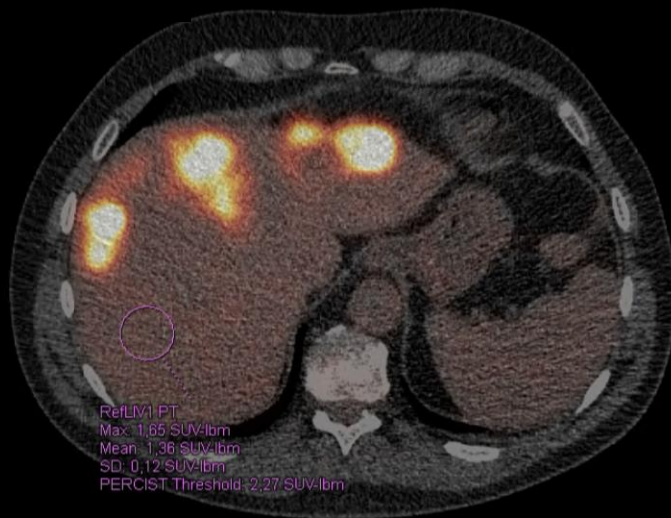

B

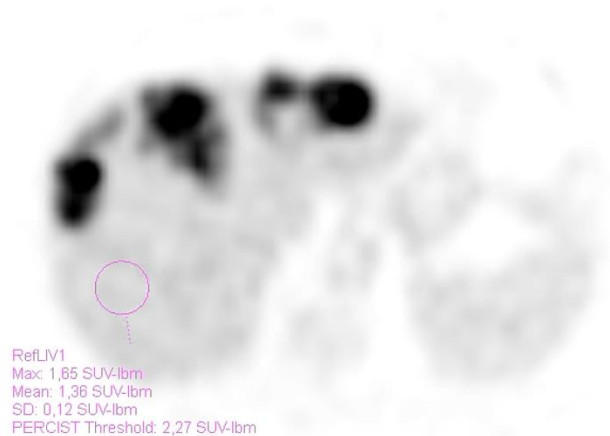

C

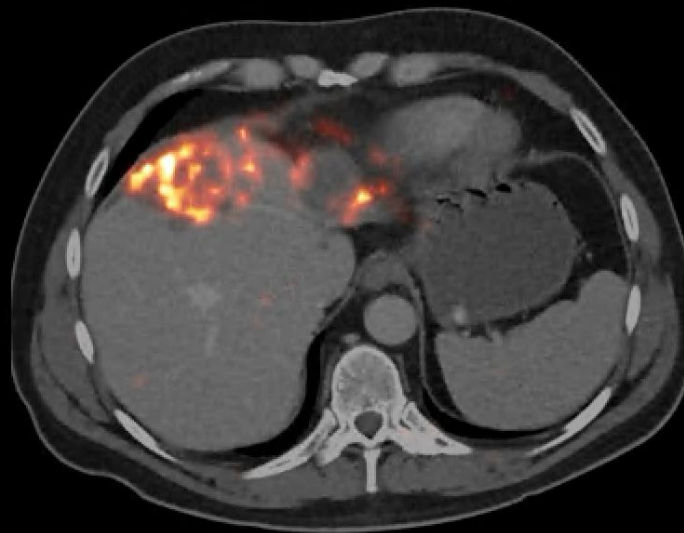

D

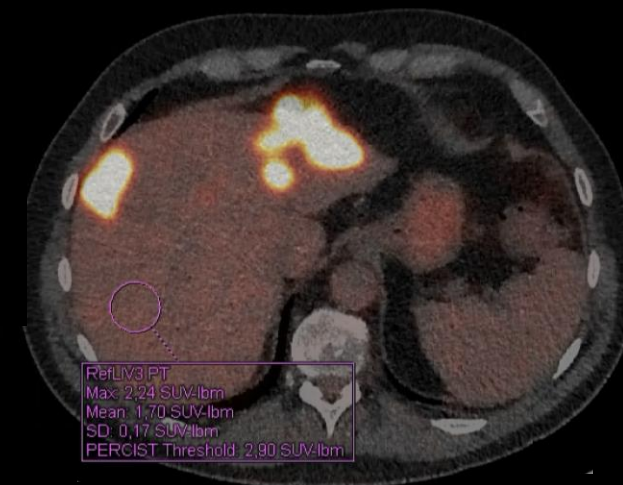

E

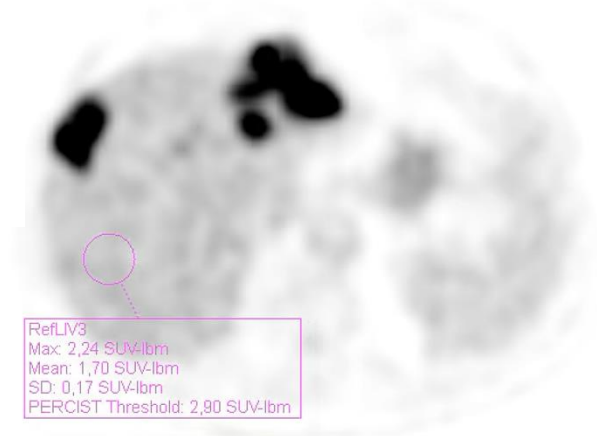

Supplement: Supplementary file 1 — Additional file 1. [file 13550_2025_1320_MOESM1_ESM.pdf]
